# The Effects of Sex-Ratio and Density on Locomotor Activity in the House Fly, *Musca domestica*

**DOI:** 10.1673/031.012.7101

**Published:** 2012-06-23

**Authors:** Simon Bahrndorff, Anders Kjærsgaard, Cino Pertoldi, Volker Loeschcke, Toke M. Schou, Henrik Skovgård, Birthe Hald

**Affiliations:** ^1^ National Food Institute, Division of Microbiology and Risk Assessment, Technical University of Denmark, Mørkhøj Bygade 19, Mørkhøj, Denmark; ^2^ Department of Bioscience, Aarhus University, Denmark; ^3^ Department of AgroEcology, Aarhus University, Denmark

**Keywords:** autocorrelation, circadian rhythm, dimorphic behavior, locomotor behavior, flies, vector

## Abstract

Although locomotor activity is involved in almost all behavioral traits, there is a lack of knowledge on what factors affect it. This study examined the effects of sex—ratio and density on the circadian rhythm of locomotor activity of adult *Musca domestica* L. (Diptera: Muscidae) using an infra—red light system. Sex—ratio significantly affected locomotor activity, increasing with the percentage of males in the vials. In accordance with other studies, males were more active than females, but the circadian rhythm of the two sexes was not constant over time and changed during the light period. There was also an effect of density on locomotor activity, where males at intermediate densities showed higher activity. Further, the predictability of the locomotor activity, estimated as the degree of autocorrelation of the activity data, increased with the number of males present in the vials both with and without the presence of females. Overall, this study demonstrates that locomotor activity in *M*. *domestica* is affected by sex—ratio and density. Furthermore, the predictability of locomotor activity is affected by both sex—ratio, density, and circadian rhythm. These results add to our understanding of the behavioral interactions between houseflies and highlight the importance of these factors when designing behavioral experiments using *M*. *domestica*.

## Introduction

Behavioral traits are important for a species' ability to disperse, mate, and search for food, and are therefore ultimately linked to the fitness of the organism (Dingemanse and Reale 2005). Complex behavioral processes include learning (Heisenberg 1998, 2003), circadian rhythm (Rosato et al. 1997; Dunlap 1999; Piccin et al. 2000; Codd et al. 2007), and courtship behavior (Partridge et al. 1987; Greenspan and Ferveur 2000).

Locomotor activity, the active movement of an animal, is an integral component of most behavioral traits and can therefore be seen as an overall indicator of the general activity of an animal. Indeed, studies have related locomotor activity in *Drosophila melanogaster* with courtship success (Partridge et al. 1987; Wisco et al. 1997). Furthermore, locomotor activity in *D*. *melanogaster* has been shown to be positively correlated with the ability to locate resources at cold temperatures (Overgaard et al. 2010), cold and hot acclimation treatment (Loeschcke and Hoffmann 2007), and oviposition success in the parasitoid wasp *Trichogramma maidis* (Bigler et al. 1988). The importance of an organism's locomotor activity in relation to stressful temperatures has also been addressed, and it has been suggested that high locomotor capacity in stressful environments is an indicator of an organisms ability to escape stressful conditions (Feder et al. 2010; Kjaersgaard et al. 2010). These observations are in agreement with the fact that locomotor activity in *Drosophila* is a highly dynamic trait that differs between populations (Gibert et al. 2001; Latimer et al. 2011).

Locomotor activity also differs between sexes, and studies have found that males of *D*. *melanogaster* show a steady and stereotyped walking pace, while females modulate their activity patterns. Locomotor activity has also been used to characterize circadian rhythm in flies. These studies have revealed very robust patterns for flies such as *Musca domestica, Calliphora vicina*, and *D*. *melanogaster* (Helfrich et al. 1985; Cymborowski et al. 1994; Helfrich-Forster 1998). The former two species show a diurnal pattern with rather constant activity, whereas *D*. *melanogaster* generally shows a bimodal pattern, with morning and evening activity peaks. However, not much is known about differences between sexes in the circadian rhythm of locomotor activity.

Despite the fact that locomotor activity is involved in many aspects of the organism's behavior and fitness, this trait has received little attention. Important questions relating to when, how and why an organism walks and how it is affected by different biological and environmental conditions still need to be addressed. For instance, most studies of circadian rhythm are based on measurements of single housed individuals and thus do not take social interactions into account, despite the potential influence on locomotor activity. In fact, many species live at high densities or in social groups most of their lives, which would make single individual observations of only minor interest when studied in isolation.

*Musca domestica* L. (Diptera: Muscidae) is an eusyanthropic fly linked to human habitats, and can be found in great numbers on farm animals, where the number of flies crawling on a single animal can regularly reach 300 flies, but can go much higher (Skovågrd and Jespersen 2000). Furthermore, it has been shown that there is sexually dimorphic behavior in physical activity and mating behavior (Ragland and Sohal 1973), and males and females also mature at a different ages (Michelsen 1960; Sacca 1964). Different sex—ratios and densities can therefore be expected to occur under natural conditions, and this makes *M*. *domestica* an interesting model organism for studying how density and sex—ratio affect locomotor activity. The aims of the present study were therefore to test for (1) differences in locomotor activity between male and female flies, (2) differences in locomotor activity between different sex— ratios, (3) differences in locomotor activity at different densities, (4) differences in diurnal and nocturnal activity, and lastly (5) differences in circadian rhythm between sexes. Based on the behavior of other insects, such as *Drosophila*, it would be expected that males show higher activity, but also a more stereotyped and predictable locomotor activity compared to females. The sex—ratios with the highest percentage of males would therefore show the highest and most predictable locomotor activity. Furthermore, density would increase locomotor activity, with males being most affected by density due to their competition for females. Lastly, we expected that locomotor activity would show a diurnal pattern with a constant activity during the light period. This was done using an infra—red light system that facilitated automated quantitative measures of locomotor activity of *M*. *domestica*.

## Materials and Methods

### Insect rearing

The house flies used were established from a Danish dairy cattle farm in 1989 and since maintained at 25 °C and 80% RH at a population size of 2000–3000 individuals. Flies were fed on water, sugar, and milk powder. Newly laid eggs were transferred to fresh larval medium consisting of wheat bran (24.6%), alfalfa (12.3%), yeast (0.6%), malted sugar (0.9%), and tap water (61.6%). Fly puparia were separated individually from the medium using a pair of sterile forceps and moved to vials that were kept at 25°C until pupation. Emerging adults were held in 250 mL vials at 25 °C with a 16:8 L:D photoperiod until used. Flies were fed *ad libitum* on sugar and water.

### Locomotor activity

Activity data were collected using a Locomotor Activity Monitor (LAM) (TriKinetics, www.trikinetics.com). This system allowed long—term recordings of flies running over days and it was therefore possible to get a very detailed picture of the insect's locomotor behavior. The system can monitor activity in 32 vials simultaneously. Each vial consisted of a 6.5 cm long capillary glass vial with an external diameter of 2.5 cm and was positioned horizontally in the LAM. Each end of the capillary glass vial was sealed with foam stoppers allowing gas exchange. Flies were provided with food during the experimental period. This was done by placing a 1.5 mL Eppendorf tube inside the foam stopper. The tube contained a sugar solution (80g/L) and was sealed with a cotton stopper. The food source was provided in the opposite end of where the light source was placed. During fly monitoring, vials were placed in a temperature cabinet (Binder, www.binder-world.com) at a temperature of 25 ±1°C. The light was provided using a LED light source giving 70 lux at the level of the flies. Parchment paper in front of the vials was used to ensure equal light intensity for all vials. Light program followed a photoperiod of 16:8 L:D (light from 07:00 to 23:00, and darkness (0 lux) from 23:00 to 07:00).

### Experimental setup

The study was divided into two experiments; in the first experiment the effects of sex and sex—ratio on locomotor activity were tested, and in the second the effect of density was tested. The flies being used were prepared according to the above procedure and subsequently divided into the locomotor activity vials, with different ratios of males and females or densities of males and females. The flies were briefly anaesthetised with CO_2_ for sex determination and were at the same time loaded into the experimental vials. This was done 24 hours prior to testing. For the group containing a ratio of males and females, three ratios were chosen, namely one male and five females, three males and three females, or five males and one female in each vial. Each sex—ratio treatment was replicated ten times, equal to a total of 30 vials or 180 flies, respectively. For the groups consisting of either males or females, three densities were chosen per sex: namely one, three, or five individuals in each vial. Each density treatment was replicated five times, equal to a total of 30 vials or 90 flies, respectively. Activity was measured as the number of times that the photocell in the middle of the vial was crossed and was recorded every five seconds. Activity for both treatment groups were measured during a full L:D cycle to establish the circadian rhythm. The vials were randomly assigned in the LAM. The flies used in the study were 48 ±24 hours of age when the experiment started, and both males and females were therefore not considered to be virgins (Murvosh et al. 1964).

### Statistics

The activity registered in each vial was divided into activity recorded during the light or dark period. The light period was composed of 11,462 recordings of five seconds each (from 07:00 to 23:00). The dark period was composed of 5761 intervals of five seconds each (from 23:00 to 07:00).

To test for differences in activity between treatments, the activity registered in each replicate were summed. This was done because the activity over time was autocorrelated within replicates and could not be taken as separate datapoints. Subsequently, activity was divided by number of flies in the vial and hours of recording. By doing so, the measure of activity was activity/hour/fly during either day or night.

Data were all tested for normality (Shapiro—Wilk), equal variances, and autocorrelation. An ANOVA was used to test for the effect of density or sex—ratio on locomotor activity, as data were normally distributed with equal variances. Differences in activity during the light and dark period and male versus female activity was tested using the Mann—Whitney U—test, since data did not meet the assumptions (normality and equal variance) for a parametric test. The raw data were smoothed for graphical presentation of circadian rhythm in locomotor activity by taking the total sum of all replicates during a five minute interval. To calculate the autocorrelation plots for each treatment, the total sum of activity for all replicates and for a period of one minute was calculated. Locomotor activity was divided by the number of flies present in the vial.

The statistical software package PAST version 1.78 (Hammer et al. 2001) was used for all the tests performed and was also used to carry out a temporal autocorrelation analysis on activity data for all the treatments and in both the light and dark period. Temporal autocorrelation was used to analyze the predictability of locomotor activity and was plotted graphically with 95% confidence intervals.

## Results

### Circadian rhythm of male and female locomotor activity

**Figure 1.**
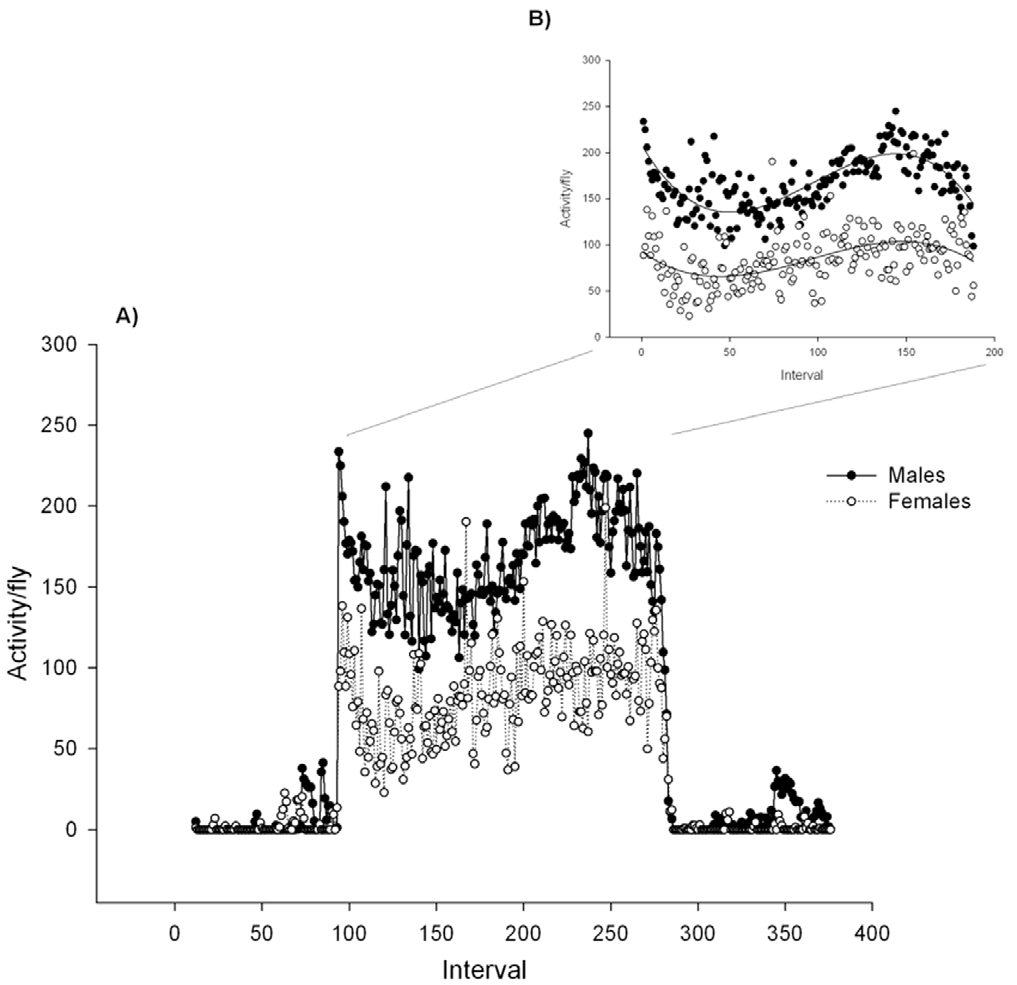
Activity per *Musca domestica* plotted for flies in vials with either three males or three females. Data is based on the sum of all replicates and has been smoothed by summing data points over five min. (A) Activity per fly across a dark:light:dark cycle, and (B) diurnal activity per fly. High quality figures are available online.

**Figure 2.**
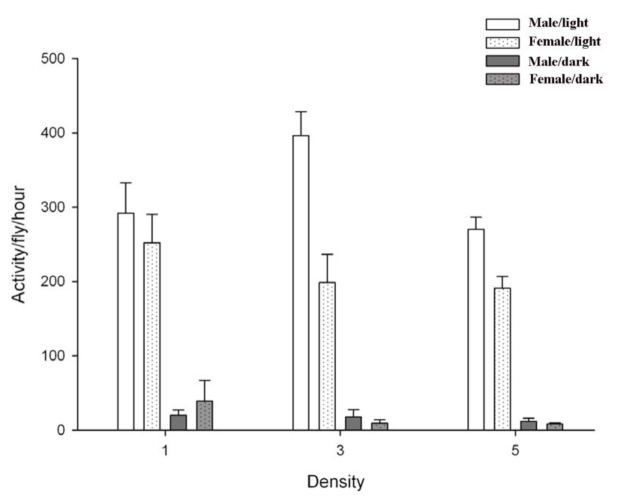
Activity per fly per hour for different densities of males and females of *Musca domestica* during the light and dark period (mean ± SE, n = 5). Activity is based on the sum of observations within replicates during 15 hours of light or seven hours of dark. High quality figures are available online.

The flies in the vials walked from one end to the other interrupted by periods of inactivity or stationary behavior. Flies did not show any preferences for one end or the other in the vials. Figure 1 shows the circadian rhythm of activity for males and females under 16:8 L:D settings. Activity changed substantially for both males and females during an L:D cycle. The total diurnal activity per hour was considerably higher than the nocturnal activity per hour for both males and females in all treatments, thus the activity data were all significantly higher (*p* < 0.01) in the light period compared to the dark period (Figure 2).

Males and females also showed different locomotor activity pattern during a L:D cycle when compared (Figure 1). Males generally showed the highest activity immediately after light was turned on, and again 12 hours after light was turned on. Females also showed this pattern but with much lower amplitude (Figure 1). The difference between sexes in circadian rhythm of activity was consistent in all density treatments. Male activity was thus higher than female activity in all treatments during the light period, but only densities of three or five flies were significantly different between sexes (*p* < 0.05). During the dark period there were no significant differences between male and female activity in all density treatments (*p* > 0.05).

### The effect of density

Males showed the highest activity at all the densities tested, with highest per individual activity at intermediate density (Figure 2). Females showed the highest activity in the vial with one female. Running a two—way ANOVA showed a significant effect of sex (*F*_1,29_ = 16.38; *P* < 0.01) on activity during the light period, but no interaction between sex and density (*F_2,29_ =* 3.289; *p* = 0.055). Subsequently, each sex was analyzed separately. Male activity in vials with three individuals had significantly higher activity compared to activity in vials with five individuals (*p* < 0.05) and one individual (*p* < 0.05). Female activity did not differ between the densities tested (*p* > 0.05). Sex, density, and the interaction of sex and density did not have an effect on nocturnal activity (*p* > 0.05).

### The effect sex—ratio

**Figure 3.**
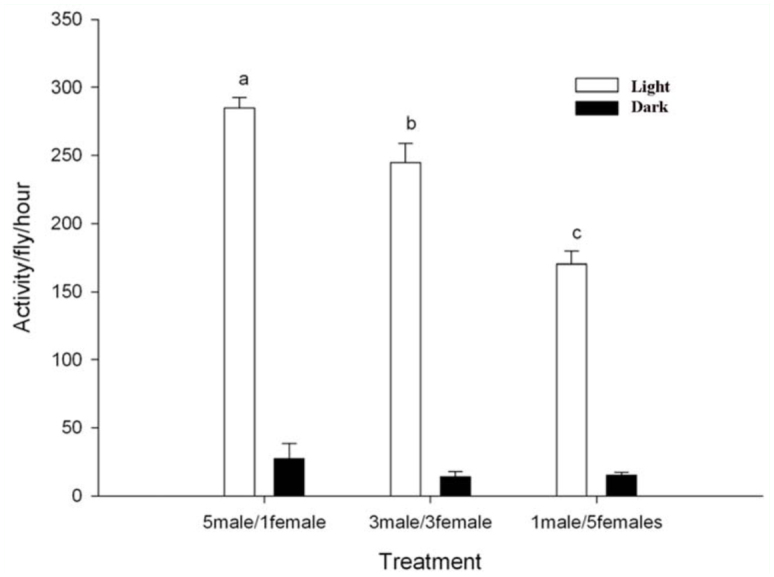
Activity per fly per hour for different sex—ratios of *Musca domestica* during the light and dark period (mean ± SE, n = 10). Activity is based on the sum of observations within replicates during 15 hours of light or seven hours of dark. High quality figures are available online.

During the light period, activity varied substantially between the different sex—ratios (*F_2,29_* = 28.36; *p* < 0.01). The highest activity was observed in the vial with five males and one female, followed by three males and three females, and one male and five females (Figure 3). However, during the dark period, activity was substantially lower and there were no significant differences between the different sex—ratios (*F_2,29_* = 0.837; *p* = 0.444) (Figure 3).

### Predictability of locomotor activity

**Figure 4.**
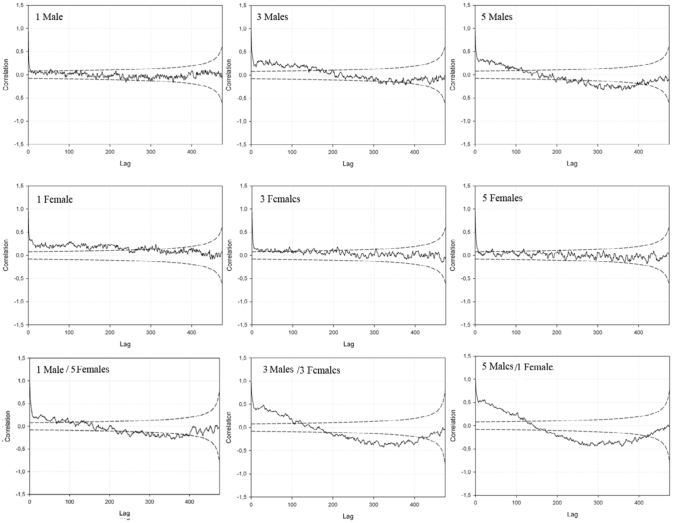
Autocorrelation plots for male and female *Musca domestica* activity at three different densities (1, 3, or 5 flies per vial) and sex—ratios (3 females and 3 males, 1 female and 5 males, 5 females and 1 male) during the light period. Autocorrelations are based on the sum of recordings for all replicates every minute per fly. Dashed lines represent the 95% confidence intervals. The values on the yaxis are the autocorrelation coefficients and time lag on the x—axis (1 unit equals 1 minute). High quality figures are available online.

The correlation between activities at different time points during the light period is shown in Figure 4. It can be seen that there was a positive autocorrelation of the locomotor activity for all the combinations of sex, sex— ratio, and density. The autocorrelation declined with different time lags when comparing the treatments (Figure 4). At a density of one male, the autocorrelation declined quickly, meaning that the activity at time *t* was not predictive of the activity at time *t + k* where *k* is a time interval. However, when looking at the higher densities of three and five individuals, there was a positive autocorrelation with a lag of up to 400 time intervals of observation. Female locomotor activity generally showed less predictability compared to males. When introducing different sex—ratios, the predictability of the locomotor activity increased with increasing number of males present in the vials (Figure 4). During the dark period there was larger variation and the degree of autocorrelation declined faster.

## Discussion

Locomotor activity is an integral component of most behavioral traits (Wisco et al. 1997). However, as pointed out by Martin (2003) not very much is known about locomotor activity *per se*, and especially changes in locomotor activity over time. Indeed this is also the case for *M. domestica* used in the present study. Even though this species is a widely used model organism and has been subjected to several behavioral studies, basic questions still need to be answered. This study addressed the effects of sex, sex—ratio, and density on locomotor activity during a full L:D cycle and found that the locomotor activity of M. *domestica* was affected by all factors, and that the activity patterns of both sexes changed during the course of the day.

The observed sexually dimorphic locomotor activity of *M. domestica* is in accordance with other studies showing that males are more active than females (Ragland and Sohal 1973; Buchan and Sohal 1981). However, due to the fact that locomotor activity was analysed during a 24–hour period and with high temporal resolution, we found that even though the flies showed a diurnal pattern, this difference was not constant and changed during the course of the day (Figure 1). Males generally showed the highest locomotor activity immediately after light was turned on, and again 12 hours after light was turned on. Females also showed this pattern, but with much lower amplitude and relatively high locomotor activity at the end of the light period compared to the males. The difference in locomotor activity between the two sexes could be due to the fact that female *M*. *domestica* are highly monogamous (Riemann et al. 1967) and may avoid male harassment by foraging and ovipositing in dim light while at the same time upholding some visual capacity. The abrupt shift in the transition from the dark to the light phase and *vice versa* in the present study may leave out some aspects of the natural temporal pattern of activity. For example *D*. *melanogaster* has been found to be most active in dim light during the day (Rieger et al. 2007), which we would not have detected in the present study. The high morning activity in males has also been found in *D*. *melanogaster* and might be explained by the eagerness of the males to find females (Helfrich-Forster 2000).

Several studies have suggested a link between mating activity and physical activity. For example, locomotor activity in different strains of *D*. *melanogaster* correlates with several indices of mating success under both dark and illuminated conditions (Kyriacou 1981; Cobb et al. 1987). Partridge et al. (1987) also found that male size affected both locomotor activity and courtship success. The link between physical activity and mating activity has also been suggested in M. *domestica*, where the rate of male wing damage owing to female avoidance behavior increases in mixed sex populations (Ragland and Sohal 1973). In the present study, we found that locomotor activity was indeed affected by sex—ratio. The results therefore support the link between activity and sexual activity.

As previously mentioned, female flies usually mate only once and afterwards try to avoid the males (Riemann et al. 1967). Female flies are also more sedentary, whereas males are more aggressive (Patterson 1957; Murvosh et al. 1964). Based on this, we hypothesized that the females would also be less predictive in their behavior and therefore show a lower degree of autocorrelation of locomotor activity. This was in fact the case, where both the vials with a high density of males and the vials with three or five males in the mixed sex—ratio treatments showed the highest positive autocorrelation. This means that males tend to show a stable locomotor activity pattern. This is in agreement with observations on males of *D*. *melanogaster*, which show a steady and stereotyped walking pace, while females modulate their activity patterns (Martin et al. 1999). Further studies are needed to clarify the sexual dimorphism in the predictability of the locomotor activity in *M*. *domestica*.

Studies looking at the effect of density on locomotor activity are scarce. Sewell (1979) found that density affected locomotor activity in *D*. *melanogaster* and showed that the effect of density was dependent on the system used to measure activity. Our results are in accordance with these findings showing an effect of density on locomotor activity for males although not for females. The change with density for males could be due to the more aggressive behavior of males (Patterson 1957; Murvosh et al. 1964). Several studies have also reported interactions in the form of attractive behavior among houseflies based on different sensory modalities. Wiesmann (1962) concluded that motionless feeding flies are the primary visual cue in food searching behavior, which was later confirmed by Collins and Bell (1996). Sex pheromones in the form of cuticular hydrocarbons also trigger excitatory responses and attraction (e.g., Richter et al. 1976) even in females (Jiang et al. 2002). Lastly, our results could be affected by multiple simultaneous crossings in the high density treatments, but multiple simultaneous crossings were considered a very rare event and therefore not likely to affect the results. Further studies are needed though to elucidate the effect of density on behavioral traits especially as many conclusions are only based on one density treatment.

The results of the present study bring us closer to a more detailed understanding of what factors affect locomotor activity of *M*. *domestica.* Furthermore, this might help improve our understanding of the behavior of flies under natural conditions. This is important as flies and especially *M*. *domestica* are natural carriers of pathogens and zoonotic agents (Banjo et al. 2005; Hald et al. 2008), and the transmission of pathogens is therefore linked to the activity and behavioral patterns of *M. domestica*. The results of the present study show that the dispersal potential might differ between sexes and/or is dependent on the density of flies and time of the day. Further studies are needed to clarify how locomotor activity in the laboratory is related to dispersal under natural conditions. Some studies have shown that laboratory assays are not always ecologically relevant and should be evaluated under field conditions (Sørensen et al. 2009; Overgaard et al. 2010).

In conclusion, the results of the present study showed that locomotor activity was affected by sex, sex—ratio, and density. Males showed higher locomotor activity and predictability than females. Density also affected locomotor activity. Lastly, locomotor activity was not constant over time and the circadian rhythm differed between sexes. These factors should therefore be taken into account when designing experiments that investigate locomotor activity in *M*. *domestica*.
